# Current status and clinical perspectives of extended reality for myoelectric prostheses: review

**DOI:** 10.3389/fbioe.2023.1334771

**Published:** 2024-01-08

**Authors:** Wei Li, Ping Shi, Sujiao Li, Hongliu Yu

**Affiliations:** ^1^ Institute of Rehabilitation Engineering and Technology, University of Shanghai for Science and Technology, Shanghai, China; ^2^ Shanghai Engineering Research Center of Assistive Devices, Shanghai, China; ^3^ Key Laboratory of Neural-Functional Information and Rehabilitation Engineering of the Ministry of Civil Affairs, Shanghai, China

**Keywords:** myoelectric prostheses, extended reality prosthetic systems, virtual reality, augmented reality, mixed reality, serious games

## Abstract

Training with “Extended Reality” or X-Reality (XR) systems can undoubtedly enhance the control of the myoelectric prostheses. However, there is no consensus on which factors improve the efficiency of skill transfer from virtual training to actual prosthesis abilities. This review examines the current status and clinical applications of XR in the field of myoelectric prosthesis training and analyses possible influences on skill migration. We have conducted a thorough search on databases in the field of prostheses using keywords such as extended reality, virtual reality and serious gaming. Our scoping review encompassed relevant applications, control methods, performance evaluation and assessment metrics. Our findings indicate that the implementation of XR technology for myoelectric rehabilitative training on prostheses provides considerable benefits. Additionally, there are numerous standardised methods available for evaluating training effectiveness. Recently, there has been a surge in the number of XR-based training tools for myoelectric prostheses, with an emphasis on user engagement and virtual training evaluation. Insufficient attention has been paid to significant limitations in the behaviour, functionality, and usage patterns of XR and myoelectric prostheses, potentially obstructing the transfer of skills and prospects for clinical application. Improvements are recommended in four critical areas: activities of daily living, training strategies, feedback, and the alignment of the virtual environment with the physical devices.

## 1 Introduction

The loss of the upper extremity is one of the most significant and destructive injuries after central nervous system damage, including spinal cord injury, stroke, and traumatic brain injury, which would cause a drastic sensory-motor deficiency, serious physical disorders, and limited daily life. Myoelectric prostheses are valuable tools for meeting the demand for functional recovery improvement of amputees, and the establishment of advanced rehabilitation techniques for upper extremity loss holds great promise for improving the quality of life of patients (Pasquina et al., 2015). However, using myoelectric prostheses in daily activities necessitates the user’s ability to produce precise and synchronized electromyography (EMG) signals, which requires extensive training and prolonged practice to achieve even minimum levels of dexterity (Resnik et al., 2012; Johnson and Mansfield, 2014). Although these prostheses have begun to implement sophisticated artificial intelligence algorithms and control schemes, the lack of appropriate training and limited integration into the activities of daily living (ADL) has contributed to high rejection rates (19%–61%) (Biddiss and Chau, 2007a; Biddiss and Chau, 2007c; Østlie et al., 2012; Salminger et al., 2022). Consequently, the efficiency of myoelectric prostheses remains a challenging problem (Biddiss and Chau, 2007b). The average waiting period from amputation to the initial prosthesis fitting is around 6 months, with no associated training provided during this time (Pezzin et al., 2004; Østlie et al., 2012; Salminger et al., 2022). Research has shown that fitting the prosthesis earlier improves compliance (Roeschlein and Domholdt, 1989). Delaying fitting would only exacerbate the user’s feelings of discomfort and hassle, highlighting the necessity for advanced pre-prosthetic training tools (McFarland et al., 2010).

Neural plasticity plays a crucial role in the utilization of myoelectric prostheses, facilitating a novel mode of coordination, which reduces phantom limb pain caused by amputation, whilst supporting long-term skill retention and transfer (Rogers et al., 2016; Preißler et al., 2017; Snow et al., 2017; Akbulut et al., 2019; Kulkarni et al., 2020). Nonetheless, it necessitates intensive muscle training to achieve control. Conventional physical therapy (CPT) is a highly repetitive exercise rehabilitation training under the supervision of doctors or therapists, which stimulates the motor nerve paths through mobilization, stretching and strengthening to enhances the control ability of the muscles of the stump (O’Keeffe, 2011; Cerritelli et al., 2021; Cao et al., 2023). This training method lacks accurate quantitative evaluation criteria for amputees. And the whole process is very arduous and monotonous. Many participants become fatigued and lose motivation, and some even completely abandon myoelectric prostheses (Resnik et al., 2012). Consequently, traditional rehabilitation training methods are difficult to help amputees to complete the target task (Stucki, 2021). There is an urgent need for a personalized, high-quality and attractive prosthetic rehabilitation training program to constantly improve equipment control, both prior to use and during the operation of the prostheses. Better training results will stem from more comprehensive, more clinical, more rewarding and entertaining myoelectric training for amputees, making rehabilitation feel less like rehabilitation. Previous studies indicate that “Extended Reality” or X-Reality (XR) systems, utilizing gamification and edutainment, can provide superior outcomes in comparison to CPT exercises. XR is a virtual environment capable of generating precise control over numerous physical factors and has been widely used in education, brain-computer interfaces and human-computer collaboration and other fields. For the training of myoelectric prostheses, XR systems have become popular tools for physical rehabilitation and motor learning, as XR helps to increase amputees’ willingness and motivation to participate in training, while also allowing for improved assessment and evaluation of progress (Radianti et al., 2020). It is a valuable resource for those seeking prostheses training, and its impact on the field is significant.

**FIGURE 1:**
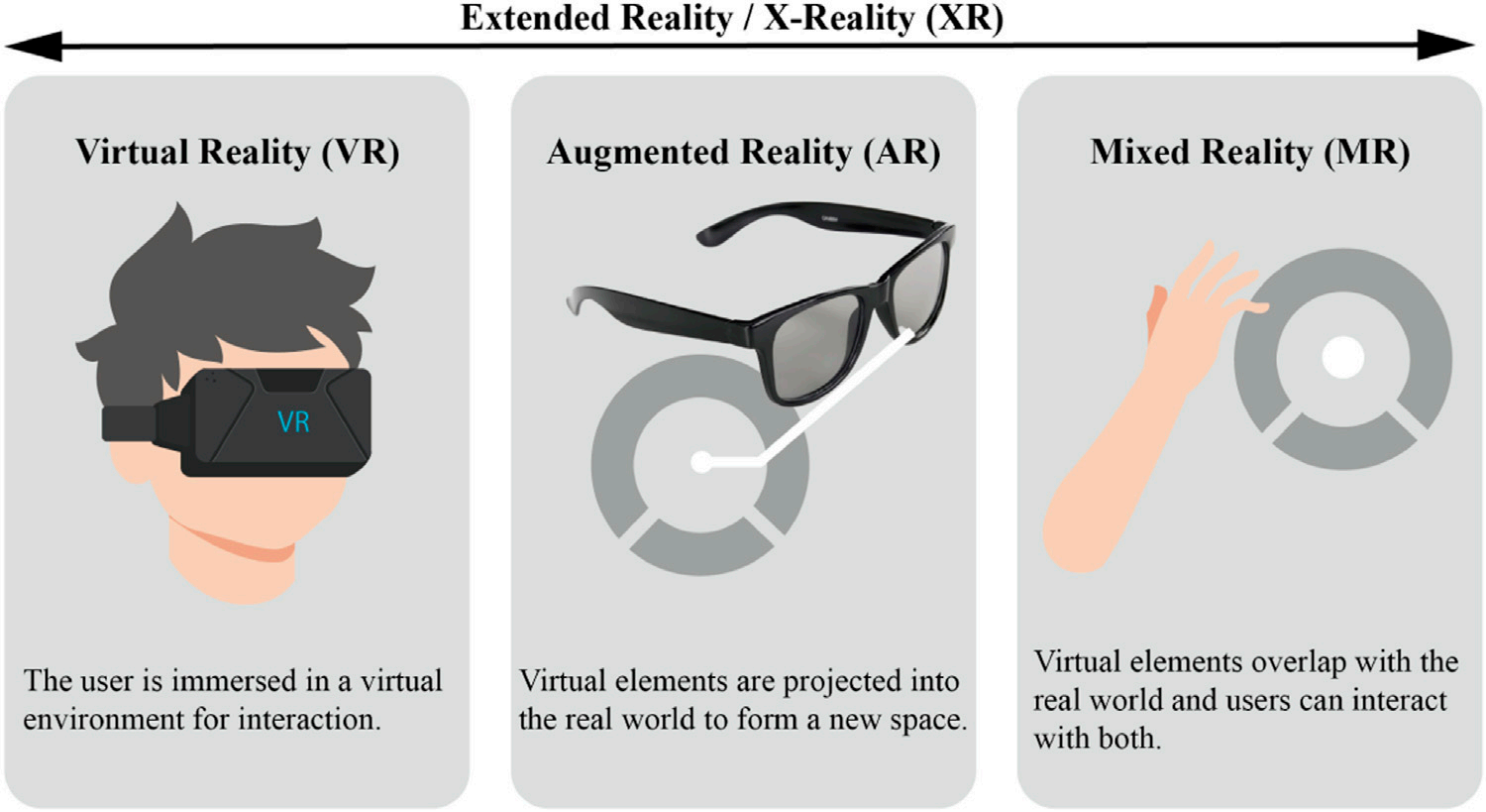
Illustrations showing the relationships and definitions between the most common realities (extended reality, virtual reality, augmented reality and mixed reality).

In this paper, the term XR in prostheses training refers to a very broad concept, which encompasses all reproduced real environments and generated virtual digital environments by computer technology and wearable devices, along with novel methods of human-computer interaction, which includes virtual reality (VR), augmented reality (AR), and mixed reality system (MR) (Figure 1). The major feature of this technology is immersion, which refers to any solution capable of delivering more immersive and captivating training experiences to patients. Apart from visual stimulus conveyed by images or videos, it may also entail other sensory stimuli, such as touch and sound. Among these technologies, VR system utilizes computer simulation to create a three-dimensional space and create a sense illusion for users, increasing the user’s sense of presence, allowing for greater interactivity within the virtual world. However, the VR system necessitates users to wear a head-mounted display with a binocular omni-orientation monitor to completely occlude the natural physical space of the surroundings, which may induce a series of problems, such as dizziness, motion sickness and other health issues. The AR system uses computer simulation to create virtual information based on physical data that is challenging to experience in real-world conditions. This virtual information is then superimposed onto the real space to generate a new picture or space that enhances the user’s visual experience and provides a sense of interaction that extends beyond reality (Hugues et al., 2011). The MR can mix virtual object information in the real space, and realize the interaction between users and virtual objects. It establishes an interactive feedback information loop between the real world, virtual space and users, enhancing realism and creating a richer experience (Flavián et al., 2019). The distinction between AR and MR is opaquer; both mix real and virtual elements and augment reality with virtual elements. The only essential difference between VR and AR (MR) is that while the former confronts the user solely in the digitally created world, the latter mixes digital with the real world (where the real world can be given either directly through transparent lenses (e.g., Microsoft HoloLens) or indirectly, through displays that stream the camera feed (e.g., Apple XR)). The XR prosthesis system refers to a virtual version of a prosthesis, built in XR environment, which does not necessarily have a control object as the prosthesis, but rather is programmed and calibrated in a manner similar to a physical prosthesis and uses simulated objects to map control commands of the EMG, allowing amputees to practice the control scheme in a well-practiced environment. XR-based rehabilitation has been proved to have some positive effects on behavior and physiology, and is very popular with elderly, Stroke, and Parkinson’s disease patients (Murray et al., 2007; Saposnik and Levin, 2011; Cao et al., 2022; Wu et al., 2022). This technology has gradually become a popular tool for clinical prostheses training, rehabilitation, and motor learning.

Compared to CPT, the XR prosthesis training have precise control over various physical factors in the environment and positively impacts the user’s physiological, psychological, and rehabilitation outcomes, thereby increasing patient motivation during therapy. Systematic data analysis can effectively record the training process and effect, provide more accurate performance evaluation methods and reduce human interpretation errors. This training approach will decrease expenses and enhance the patient’s innate drive, thus augmenting their commitment towards neuromuscular rehabilitation training. (Garske et al., 2021). shows that there are a large number of prosthetic training software based on serious games, which focus more on improving engagement and muscle training, without paying attention to the importance of skill transfer. (Gaballa et al., 2022). introduces the existing virtual prosthetic training technology and the user evaluation procedure to ensure the practicability in the clinical environment, and identify obstacles in technology, human factors, clinical and management levels, economy, and suggest possible pathways to deployment for successful clinical adoption in the future. (Toledo-Peral et al., 2022). analyzed the application of VR/AR in motor neurorehabilitation after stroke/amputation, including the scope of application, characteristics, target anatomical region, how to use, signal processing methods and hardware. Prosthesis training using XR techniques for upper limb amputees has the potential to enhance competency or speed up the learning process for acquiring the skill; however, there is no consensus on which factors are crucial in the transfer of skills from virtual training to actual prosthetic competence. In light of the above, the current review focus on four critical areas:

• Components and available cases of the XR prosthetic system,
• Training methods and evaluation metrics of the XR Prosthetic System compared to other rehabilitation protocols,
• What are the technical limitations and barriers in the process of skills transfer?
• Possible deployment pathways for future successful clinical applications.

We surveyed papers dealing with upper limb prosthesis training or assessment with the assistance of the XR environment and using EMG signals as input. Relevant papers were identified during the literature survey and enhanced by systematic searches using PubMed, Web of Science, Science Direct, IEEE Xplore, Google Scholar and SCOPUS databases. Based on a summary of existing XR prosthetic systems, with associated control methods, assessment methods and evaluation metrics, and comparing the application of prosthetic hands, this review analyses the characteristics and shortcomings of the existing systems in the process of skill transfer from virtual training to actual prosthetic ability in four aspects: ADL, training methods, feedback, the relationship between the virtual environment and the physical device.

## 2 The existing XR systems for upper extremity prostheses

The two most important aspects of XR for myoelectric prosthetic hand are the user interface and myoelectric control.

### 2.1 User interface

The XR prosthesis system offers an interactive environment that enables users to repeat various actions. Most of the time, this type of interface works to immerse users in a virtual environment and perform virtual actions using electromyography control, which gives users the sensation of experiencing a similar movement in reality (Woodward et al., 2017). According to the immersion level applied to XR, it can be categorized as nonimmersive XR and immersive XR (Sveistrup, 2004). Nonimmersive XR involves interactions between an environment and players via a computer monitor or non-HMD display, maintaining a safe distance between participants and the game (Bevilacqua et al., 2019). Immersive refers to the utilization of various head-mounted displays (such as Occulus Rift Headset, HTC VIVE Pro, Google Glass, Meta Glass, and Microsoft Hololens), which are connected to the human body to interact with the game (Narayanasamy et al., 2006; Lu et al., 2012) clarify that the user interface of XR systems can be categorized into two types: serious games and simulation tasks. Serious games replace prostheses with gamification elements in fictional scenarios, maps EMG control commands with specific game goals, which is able to provide a variety of challenges, increase the enjoyment of training, and optimize the learning process. Conversely, simulation tasks generally involve recreating a prosthetic-like control object and duplicate the controls in real-world scenarios, requiring standard operating procedures and lacking in entertainment.

**FIGURE 2:**
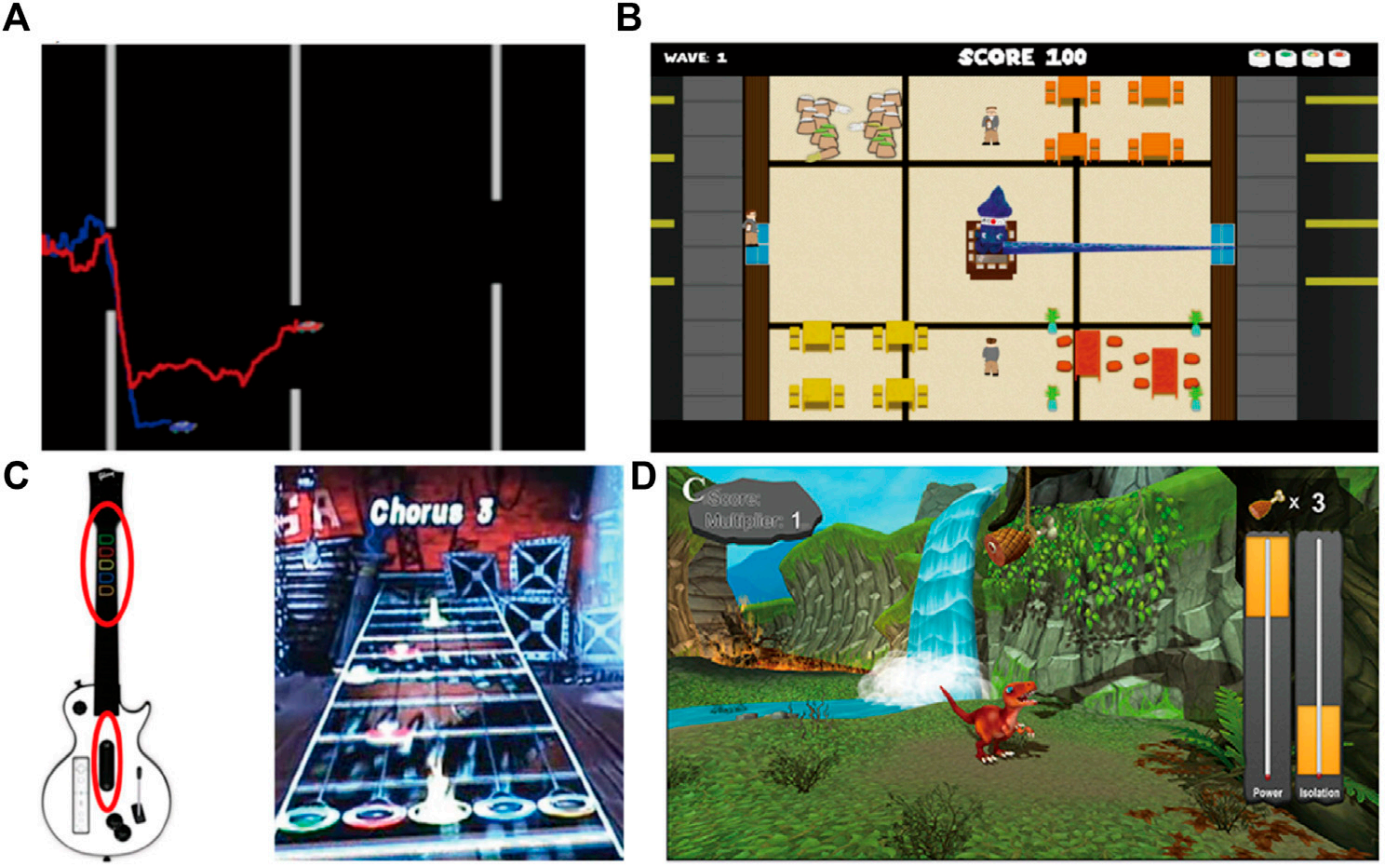
Serious game: **(A)** MyoBoy; **(B)** Sushi Slap; **(C)** Air Guitar Hero; and **(D)** Dino-Feast.

Since the early 1990s, serious games have been researched for prostheses control training (Lovely et al., 1990), which is a video game with an explicit and carefully thought-out educational purpose and intended to impart certain knowledge or skills to users (Graafland et al., 2012; Laamarti et al., 2014). As a virtual training system, serious games can increase patients’ motivation, improve muscle coordination, and ultimately augment electromyography control ability (see Table 1) (Clingman and Pidcoe, 2014). MyoBoy (Figure 2A) and PAULA or Virtu Limb™ are mature computer-based electromyography training systems. These systems use the subject’s flexor and extensor muscles to improve electromyography control. Patient feedback has indicated that the current commercial method, which depends on basic graphic representations of EMG, is less motivating and satisfying than the training system that is reliant on serious games (Prahm et al., 2017a). Several serious games based on traditional game design, such as Pong (de la Rosa et al., 2008), Flappy Bird (Radhakrishnan et al., 2019), Space Invaders (Radhakrishnan et al., 2019), SuperTuxKart (Prahm et al., 2017b), Sushi Slap (Smith et al., 2018b; Smith et al., 2018a) (Figure 2B), Crazy Meteor (Smith et al., 2018b; Smith et al., 2018a), Dog Jump/Beeline Border Collie (Smith et al., 2018b; Smith et al., 2018a), Crate Whacke (Hashim et al., 2021a), Race the Sun (Hashim et al., 2021a), Fruit Ninja (Hashim et al., 2021a), and Kaiju Carnag (Hashim et al., 2021a), employ a method similar to the user’s control of a physical prosthetic hand, which not only repeatedly activates the flexor and extensor muscles, but also instigates the random training of joint or continuous muscle contraction. This approach provides an ideal training method for direct control (DC), while also enhancing the motivation and adherence of the amputation rehabilitation plan. Rhythm games and car racing games, such as Air Guitar Hero (Armiger and Vogelstein, 2008) (Figure 2C), MyoBeatz (Prahm et al., 2019a,8), UpBeat(Melero et al., 2019), and Sonic Racing (Martinez-Luna et al., 2020), incorporate sound feedback into traditional gameplay, which are valuable for early-stage rehabilitation and provide solid starting points for the inclusion of feedback (Prahm et al., 2018). Mobile phone games, such as such as Volcanic Crush incorporate based dual-site muscle activation, Dino Spirit and Dino Feast (Figure 2D) involving sequential and proportional movement control, and Dino Claw with 3-D movement control, create more opportunities for myoelectric training outside the clinical environment, which overcome logistical, financial and geographical barriers to users, and increase training motivation (Winslow et al., 2018). For improving the training performance, serious games have the following characteristics: 1) The subjects focus on the screen and can find the best training scheme to the challenge through implicit learning without clear prompts (Kristoffersen et al., 2021); 2) Tasks of varying difficulty levels can be provided to enhance the interest and motivation of the subjects as well as extend their training time (Rahmani and Boren, 2012); 3) Remote personalized guidance can be provided by therapists or doctors (Holden, 2005); 4) Real-time feedback can be incorporated to optimize the training effect (van Diest et al., 2013). Serious games offer a simplified myoelectric control interface displayed on a computer screen. While unable to display quantitative results, it provides direct control of a limited set of muscles with intuitive functionality. Its usefulness is limited to early-stage rehabilitation and does not induce changes in muscle performance.

**TABLE 1:** Detailed categorization of the serious game.

Program (Genre)	Task	Feedback mechanism	Control strategy	Performance metrics	Skill transfer
Myoboy	Abstact task	Traditional Media	DC		No
Air-Guitar Hero (rhythm game)	Abstact task	Traditional Media	ML	score	No
WiiEMG (sports game)	Abstact task	Traditional Media	ML	Time, accuracy	No
Sonic Racing (racing game)	Abstact task	Traditional Media	DC	Time	No
MyoBox (dexterity game)	Abstact task	Traditional Media	ML	Separability, consistency, variability	Yes
MyoBeatz (rhythm game)	Abstact task	Traditional Media	DC	SUS, proportional muscle activation	No
Falling of Momo (vertical scroller)	Abstact task	Traditional Media	DC	UES, IMI, SUS	No
Volcanic Crush (reaction game)	Abstact task	Traditional Media	DC	UES, IMI, SUS	No
Dino Sprint (endless runner)	Abstact task	Traditional Media	DC	UES, IMI, SUS	No
ino Feast (dexterity game)	Abstact task	Traditional Media	DC	UES, IMI, SUS	No
Breakout-EMG (arcade game)	Abstact task	Traditional Media	DC	Accuracy	Yes
Crossbow Game	Posture reproduction	VR	ML	Postures completed score	No
UpBeat (rhythm game)	Posture reproduction	AR	ML	Gesture completion, muscle activation	No
MyoTrain	Posture reproduction	Traditional Media	ML	Accuracy	No

**FIGURE 3:**
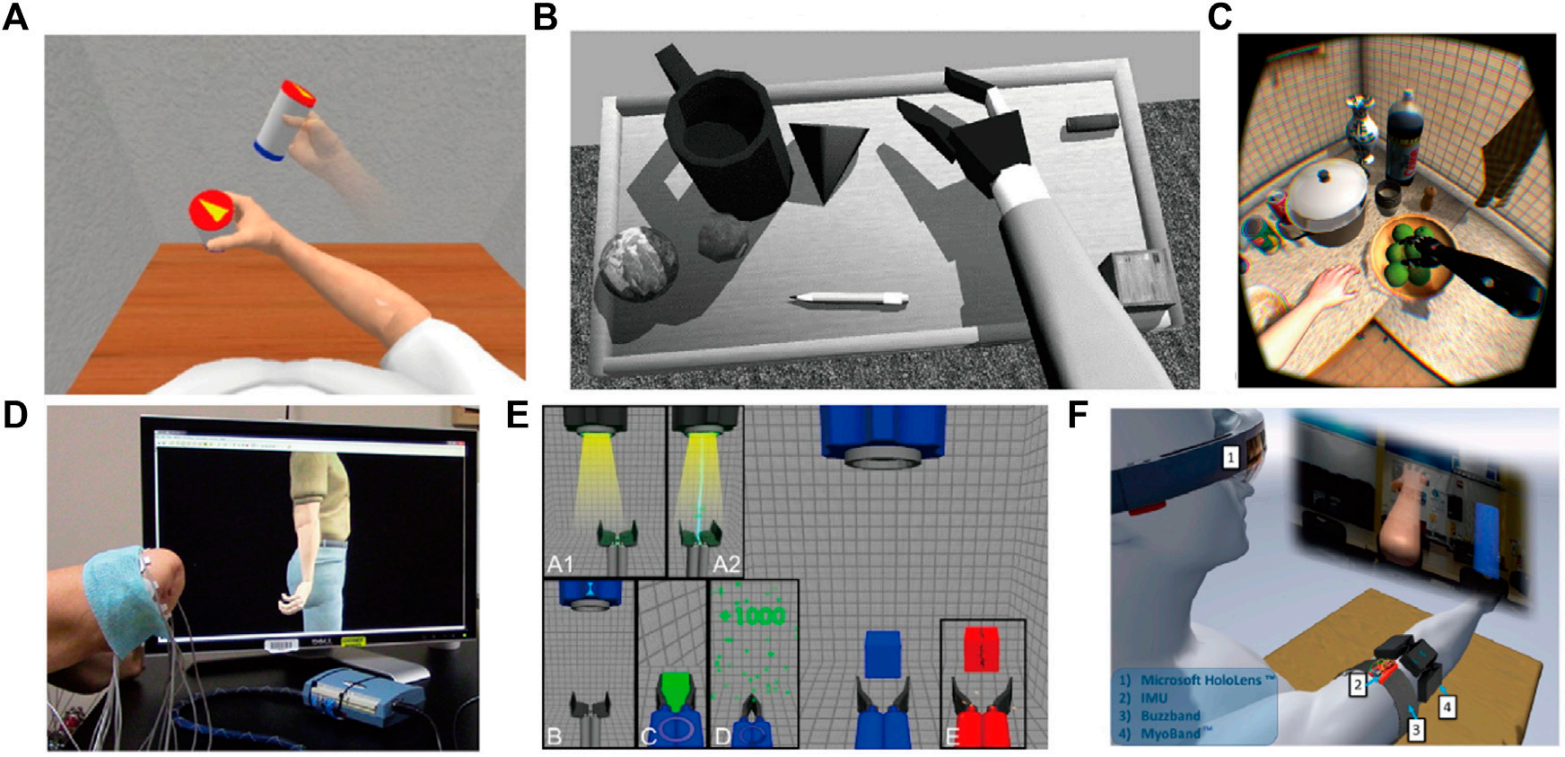
Simulation tasks: **(A)** VRd testing environment; **(B)** Prostheses simulator; **(C)** Exploration; **(D)** VIE; **(E)** Catching simulator Prostheses Gripper; and **(F)** Mixed reality training.

Simulation tasks are typically presented in either the third-person perspective or the first-person perspective. The former aids the user’s spatial perception, while the latter can enhance the user’s sense of interaction with the virtual object. Both perspectives offer distinct benefits, and align with the desired functionality of myoelectric rehabilitation systems. Generally, simulation tasks involve posture reproduction tasks, which necessitate following typical operating procedures and assessment indicators but lack entertainment value (see Table 2). XR systems based on simple simulation tasks, such as UVa-NTS platform (de la Rosa et al., 2009), Virtual training environment (Cavalcante et al., 2021), VRd testing environment (Blana et al., 2016) (Figure 3A), Training environment (Al-Jumaily and Olivares, 2009), and Virtual model (Muri et al., 2013), provide a solid research foundation for making virtual prosthetic systems and training amputees. Simulation tasks combined with standard training schemes, such as Virtual box and beans test (Prahm et al., 2019b), Virtual box and blocks test (Hashim et al., 2021b), Virtual rehabilitation training tool (Dhawan et al., 2019,8), Virtual Therapy Arm (VITA) (Nissler et al., 2019), AR prostheses simulator (Kenedy Lopes, 2012), Virtual training system (Nakamura et al., 2017), Performance assessment (Hargrove et al., 2007), Prostheses simulator (Lambrecht et al., 2011) (Figure 3B), and Virtual reality environment System (Resnik et al., 2011), have shown promise as a tool for developing and evaluating control methods by enhancing and refining particular skills. XR systems based on complex environment, such as Exploration (Phelan et al., 2015) (Figure 3C), Virtual simulation (Soares et al., 2003), and HoloPHAM (Sharma et al., 2019), have created virtual environments that are more suitable for daily life, which can satisfy users’ sense of immersiveness. These systems are being studied how to best assess the impact and accuracy of such environment. Open source systems, such as VIE (Perry et al., 2018) (Figure 3D) and Musculoskeletal Modelling Software (MSMS) (Davoodi and Loeb, 2012), prove that amputees can effectively learn the EMG contraction mode, provide effective training platforms based on machine learning (ML) control, and make it possible for different research groups to develop effective and unified training methods. Systems based on virtual prosthetics, including Catching simulator (van Dijk et al., 2016b), Catching simulator Prostheses Gripper (Kristoffersen et al., 2021) (Figure 3E), and MSMS, have demonstrated the transfer effect and existing deficiencies from virtual prosthetics to physical prosthetics. Imitation-oriented XR exercises can produce lower practice variability, and assist with movement learning by promoting consistent movements through accurate repetition. ADL-oriented XR could elicite stronger muscle activity and movement variations. The combined design appears to yield superior training outcomes. Several XR prosthetic systems, such as ARlimb (Boschmann et al., 2016; Boschmann et al., 2021), AR prostheses simulator, and Mixed reality training (Sharma et al., 2018) (Figure 3F), illustrate the differences between AR/MR and VR. However, the systems are not compared to one another.

**TABLE 2:** Detailed categorization of the simulation tasks.

Program (Genre)	Task	Feedback mechanism	Control strategy	Evaluation procedure	Performance metrics	Skill transfer
UVa-NTS platform	Abstact task	Traditional Media	DC		Success rate, time	No
PAULA	Abstact task	Traditional Media	DC		Velocity, error	No
Virtual training environment	ADL	VR	ML	BBT	Score	No
Mixed reality training	ADL	MR	ML	PHAM	time	No
Virtual box and blocks test	ADL	VR	DC	BBT	Score	Yes
Virtual box and beans test	ADL	Traditional Media	ML	BBT	IMI	No
Virtual Therapy Arm	ADL	VR	ML	BBT	Score	No
Exploration	ADL	VR	ML		Score	No
Catching simulator	ADL	Traditional Media	DC, ML		Score	Yes
Performance assessment	ADL	Traditional Media	ML	CRT	Accuracy, pin time, Classification errors	No
VR evaluation environment	Posture reproduction	VR	ML		Accuracy	No
ARlimb	Posture reproduction	AR	ML	CRT	Accuracy	Yes
Training platform	Posture reproduction	Traditional Media	ML		Accuracy	Yes
HoloPHAM	ADL	MR		CRT, PHAM		No

### 2.2 Myoelectric control

Currently, the mainstream myoelectric control methods ARE DC and ML. The DC primarily employs EMG signals from two muscle groups to control all possible grasping modes, including on/off control, sequence control, and mode switching control. This control requires users to actively switch among multiple degrees of freedom (DoFs). Due to its ease and implementation, DC is the most cfrequently utilized control approach in commercial prostheses and XR systems. Unlike DC, ML uses electrodes with more than two to measure EMG on multiple muscles in the stump, and calculates EMG features that can be mapped to the input of the learning algorithm for prosthetic control commands. This in turn enables users to generate a potentially larger range of control commands. ML can realize simultaneous control of multiple DoFs by using muscle contraction mode, which aligns more closely with the neural pathway of natural human control and can also minimize compensatory movements of the trunk and shoulder. ML control can reveal the full potential of prostheses, potentially improving prosthesis function and reducing the burden on upper extremity amputees.

Unlike the DC based XR system, games using ML control, like Crossbow Game, are not designed specifically for training users. In these systems, users cannot distinguish which type of muscle contraction corresponds to which type of motion. Thus, they can explore every possible muscle contraction that can be performed throughout training, resulting in both muscle contraction and algorithm adapting to one another to achieve better myoelectric control (Kristoffersen et al., 2021; P, 2016). The Rehabilitation Institute of Chicago proposed that the XR system based on ML entails a sophisticated training process comprising of four distinct stages: Conceptual Training (teaching the principle of system and determining which movements would be used to achieve better control), Control training (providing guidance when learning to use the system), Function use training (testing prostheses used in daily life activities), Prostheses recalibration training (teaching how to maintain system performance in daily use) (Simon et al., 2012). Systems based on adaptive algorithms, such as VR evaluation environment and Virtual box and blocks test, can successfully prevent XR system performance decline during extended training session (Lambrecht et al., 2011). Most papers surprisingly lack detailed descriptions of processing algorithms. Presently, the utilization and processing of EMG signals in the realm of upper extremity prostheses remain scattered and heterogeneous, lacking consensus on the selection methods of signal processing, classification algorithms, and performance evaluation. We suggest it is crucial to elucidate these concepts as one of the technical guidelines for fostering consistency within the proposed protocols.

## 3 Clinical outcome assessments and performance metrics

Effective evaluation methods can enhance the assessment of muscle control ability and the efficacy of the XR prosthetic system, thereby facilitating amputee rehabilitation training. The clinical assessment of prosthetic user outcomes are typically assessed through subjective patient-report outcome measures and objective performance-based outcome tests (Wang et al., 2018). The use of subjective patient-report outcome measures allows for the disclosure of subjective details regarding improvements in daily activities, an assessment of user satisfaction with the device, and the evaluation of impacts on life quality. This measurement is preferred because it provides insight into the subjective information regarding the training effect’s improvement and the evaluation of the user’s satisfaction with the system. Meanwhile, the objective performance based measurement utilizing standardized procedures is able to evaluate system performance, provide quantitative results that are objective, unbiased, and repeatable, and effectively aid both the therapist and user in improving training. While the subjective patient-report test offers a detailed understanding of the patient’s experience with the device, it may be biased and influenced by their memory of past events and perspectives. An objective, performance-based measure accounts for these issues but does not address the user’s attitude towards the device. In other words, a testing methodology that relies solely on performance-based measures disregards the patient experience, potentially overlooking long-term concerns. Therefore, to ensure effectiveness and suitability upon deployment, clinical rehabilitation tools must undergo comprehensive testing using both objective performance-based measures and subjective patient reports.

### 3.1 Subjective patient-report outcome measures

Intrinsic Motivation Inventory (IMI), System Usability Scale (SUS), User Evaluation Survey (UES), and NASA Task Load Index are four prominent measures in subjective patient-report outcome measures. IMI is composed of several subscales, which mainly rate the enjoyment, perceived choice, perceived competence and immersion of XR system to evaluate the experience of playing video games (Anderson and Bischof, 2014; Hashim et al., 2021a). SUS is a questionnaire with 10 items, involving the stations, overall game experience, virtual reality experience and all session experience, which is used for quick usability evaluation across multiple domains (Bangor et al., 2008; Dawson et al., 2012). UES mainly scores the game input, control, motivation and fun, including 1) rating the game, 2) rating the input 3) rating the control methods, 4) rating the EMG assessment, and 5) determining the attractive elements (Prahm et al., 2017b; Prahm et al., 2017c). The NASA Task Load Index has been utilized multiple times with upper extremity prostheses, which contains various questions to evaluate mental and physical demand, temporal demand, task performance, effort, and frustration (Osborn et al., 2021; Chappell et al., 2022; Parr et al., 2023).

### 3.2 Objective performance-based outcome measures

In the designing of XR prostheses training system, therapists utilize various training tools to restore control of the residual limb during daily activities. Some of these tools have undergone clinical verification while others are mentioned in literature (Lindner et al., 2010). Clinical outcome assessments (COAs) are employed to assess the progress of individual rehabilitation or training through XR system. Research has demonstrated that motor control learning is highly specific. Effective evaluation methods can provide more accurate assessments of muscle control ability and the effectiveness of XR system, and can promote the rehabilitation training for amputees (Giboin et al., 2015; van Dijk et al., 2016b). Consequently, selecting appropriate training activities to assist prosthetic users in returning to their regular routines is critical. While physical prosthetic devices form the basis of most of these methods, training in virtual environments has emerged as an effective means of assessing patients’ performance during daily living tasks. After reviewing the available literature, this paper outlines 14 frequently utilized clinical outcome measures for the performance-based assessment of residual limb training (Table 3).

**TABLE 3:** Commonly used clinical outcome indicators.

Performance metrics	Procedure	Properties	Deficiencies
Motion test	Execute the appropriate movement following the virtual prostheses	Investigated changes in EMG levels	Oversimplified
TAC test	Control the virtual prostheses to move to the target posture	Adjust the speed of the virtual prostheses according to the muscle contraction	Interaction space is a virtual environment rather than a physical environment
BBT	Move the block from one side of the box to the other	Allows continuous estimation of single-finger activation and incremental learning	Focus on a limited number of DoFs only
NHPT	Pick up the pegs, put them into the hole on the board, and remove it	Ability to perform flexibility testing	Relatively simple and short
CRT	Move the clothespins from the horizontal bar to the vertical bar	Perform repetitive coordinated stretching and grasping movements	Results scoring without corresponding compensatory movement
Task tests	Simulation of prosthetic gripping tasks	Improved performance of transfer from virtual space to prostheses	Differences between virtual space and actual tasks lead to errors
JHFT	7 ADL tasks	Simulate ADL corresponding to prostheses use in daily life	Training differences between the virtual and real environment
AM-ULA	18 ADL tasks that can be divided into subtasks	Assessment of awkwardness and compensatory exercise	Training differences between the virtual and real environment
CAPPFUL	11 ADL tasks	Assesses the ability, time and quality to complete activities	Training differences between the virtual and real environment
ACMC	30 functional hand movements that can be categorized into 4 hand use	Measuring the ability to operate prostheses while performing ordinary life activities	Influenced by a relatively large subjective component
ARAT	19 arm function assessment tasks	Objects to be moved to shelves of different heights	Influenced by subjective components
SHAP	14 ADLs and 12 additional object transfer tasks	Assesses ability to execute specific grips	lengthy and tiring
AHAP	26 grasping tasks	Replicability using publicly available Yale-CMU-Berkeley objects	Converts complex tasks into simple grasping tasks
PHAM	Manipulate a group of objects by grasping them and changing their position	Ability to monitor gesture completion rates and consider compensatory movements	Lack of comprehensive quantitative assessment methods

**FIGURE 4:**
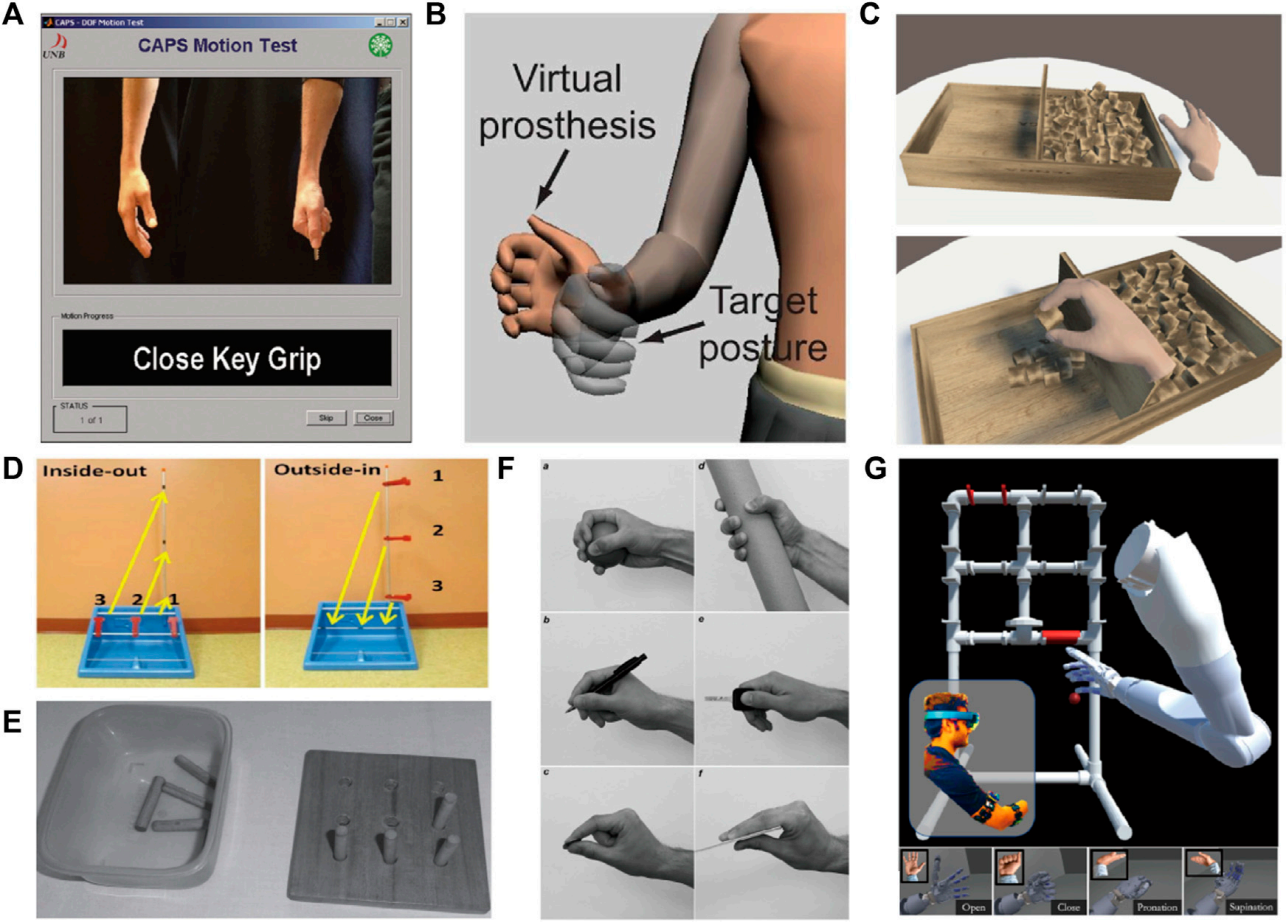
Objective performance-based outcome tests: **(A)** Motion Test; **(B)** TAC; **(C)** BBT; **(D)** CRT; **(E)** NHPT; **(F)** SHAP; and **(G)** PHAM.

For Motion Test (Figure 4A), participants received instructions to follow the motion prompts while observing the virtual prostheses that decoded their movements. This test aimed to investigate changes in EMG levels, but it oversimplified the study by not examining changes in muscle function levels (Kuiken, 2009; Kristoffersen et al., 2020; Portnova-Fahreeva et al., 2023).

Unlike the Motion Test, the Target Achievement Control (TAC) (Figure 4B) test enables subjects to move the virtual prostheses at a slow or fast pace based on their muscle contraction intensity (Simon et al., 2011). Assessment criteria consist of Test Complexity, Movement Distance, Target Width, Dwell Time and Trial Timeout. Misclassification may aid in completing the motion gradually. One limitation of TAC testing is the absence of interaction between the subjects and the virtual environment (Boschmann et al., 2016; Hargrove et al., 2018; Woodward and Hargrove, 2019).

The Box And Block Test (BBT) (Figure 4C) instructs subjects to move blocks from one compartment of the box to another as much as possible within 60 s (Mathiowetz et al., 1985a). This assessment evaluates the user’s capability to perform fundamental actions using a prosthetic device. However, there is not an evaluation test for proportional force control (Hebert and Lewicke, 2012; Kontson et al., 2017). To enhance the system’s modular features, the BBT incorporates everyday virtual daily-living activities scenes, such as the living room and kitchen (Nissler et al., 2019). In this setting, it can cause alterations in other areas of the participant’s body and handle items of varying elevations.

Similar to BBT, the Nine-Hole Peg Test (NHPT) (Figure 4E) requires subjects to insert and remove wooden pegs into and out of holes on a board, with scoring based on the time and speed required to complete the task (Mathiowetz et al., 1985b; Oxford Grice et al., 2003; Kristoffersen et al., 2021).

The Clothespin-Relocation Test (CRT) (Figure 4D) is an established tool for testing upper limb flexibility, by assessing the time required to reposition the three pins of the Rolyan Graded Pinch Exerciser system from the horizontal bar to the vertical bar (Hussaini and Kyberd, 2017; Kyberd et al., 2018; Hussaini et al., 2019). It realizes precise myoelectric control and coordinated movement of the upper limb joint through repeated coordinated reaching and grasping movements, and repositioning the clothespin in space.

Task Tests refers to task-specific tests such as grasping, interception, tracking, matching, and object recognition (Bouwsema et al., 2014; van Dijk et al., 2016b; Manero et al., 2019). This test can enhance the information related to ADL in a game-relevant way, and improve the performance of XR transfer to the prostheses. The limitation of this test lies in the design of virtual tasks, and it is impossible to calculate the error amount and solution space related to each goal.

The Jebsen-Taylor Test of Hand Function (JHFT) (Davis Sears and Chung, 2010; Wang et al., 2018), the Activities Measure for Upper Limb Amputees (AM-ULA) (Resnik et al., 2013) and the Capacity Assessment of Prosthetic Performance for the Upper Limb (CAPPFUL) (Kearns et al., 2018) are designed to train or assess various unimanual hand functions required for ADLs with corresponding objects. These three COAs consist of 7, 18, and 11 ADLs, respectively, which are used to assess the ability to perform activities, completion time and movement quality. JHFT is a series of standardized activities, including writing a sentence, page turning, stacking checkers, simulated feeding, picking up/lifting large objects, picking up/lifting heavy objects and picking up/lifting small objects. During training, the completion of these activities is graded by time, with a maximum time limit of 120 s. AM-ULA tasks include combing hair, putting on and taking off clothes, buttoning a shirt, zipping a jacket, tying socks, tying shoes, pouring soda, turning a doorknob, hammering, folding a towel, using a cup, fork, spoon, scissors, and telephone, writing a word, reaching overhead, etc. Each task is further divided into subtasks according to the steps required to complete the task. Task scoring is based on the extent of subtask completion, speed of completion, quality of movement, grip control and prosthetic skills, and independence.

The Assessment for Capacity of Myoelectric Control (ACMC) is an observational assessment designed to measure prosthetic control of ADLs (Hermansson et al., 2004). It consists of 32 functional hand movements, which are divided into 4 categories of hand use: gripping, holding, releasing, and coordinating. In addition, it uses a 4-category scale to identify and evaluate hand movements and judge the ability of subjects to perform spontaneous movements. In all evaluations, only ACMC has been clinically shown to have good test-retest reliability for upper extremity prostheses (Hermansson et al., 2006).

The Action Research Arm Test (ARAT) consists of 19 tasks, which are divided into 4 categories: grasp, grip, pinch, and gross movement (Fitts, 1954). Meanwhile, the test requires the subjects to move objects to different heights of shelves, manipulate common objects, such as washers and blocks, and perform ADLs, such as pouring water into a glass. Some tasks also assess the arm range of motion.

Southampton Hand Assessment Protocol (SHAP) (Figure 4F) is one of the most detailed hand function assessment tools available. It consists of 26 separate tasks, including six grip types (spherical, tripod, tip, power, lateral, and extension), which can be divided into abstract object processing (light/heavy sphere, tripod, power, lateral, tip and extension) and ADLs (pick up coins, undo buttons, food cutting, page turning, remove jar lid, pour water from jug and carton, move a full jar, an empty tin, and a tray, rotate a key, screw, and door handle, open/close a zip) (Bouwsema et al., 2012; Burgerhof et al., 2017). It mainly quantifies the time required to perform the task, regardless of how the task is performed. It is tedious and exhausting for amputees with limited abilities (Vasluian et al., 2014; Kyberd, 2017; Kristoffersen et al., 2021).

The Anthropomorphic Hand Assessment Protocol (AHAP) is a digital standard to quantify the ability of prostheses to perform daily grasping, which is divided into 26 tasks (Llop-Harillo et al., 2019). According to the kinematic structure of the hand and grasp frequency of ADLs, these tasks are divided into eight grasp types [pulp pinch (PP), lateral pinch (LP), diagonal volar grip (DVG), cylindrical grip (CG), extension grip (EG), tripod pinch (TP), spherical grip (SG) and hook grip (H)] and two non-grasping postures [platform (P) and index pointing/pressing (IP)]. To account for changes in object size, shape, weight, texture, and stiffness during human-environment interaction, each grip type selects three different objects from the YCB suite to achieve reproducibility (Llop-Harillo et al., 2022).

Prosthetic Hand Assessment Measure (PHAM) (Figure 4G) is a standard for upper limb amputees to quantitatively evaluate a series of operational tasks related to object manipulation (e.g., water, pencil, coin, and power), focusing on monitoring gesture completion rates and compensatory movements (Hunt et al., 2017; Sharma et al., 2019). In PHAM protocol, users need to grasp objects with specific gestures and change their position in the frame to manipulate a group of objects within the physical frame (Melero et al., 2019).

### 3.3 Performance metrics

For DC, the training focuses on two muscles that are independent of each other in terms of contraction function, as well as execution of the mode switching command. For ML, the key point is to adapt several muscle groups to produce EMG patterns that can separate different actions and repeat the same action. Myoelectric control depends on each muscle playing its role during training, so using XR system for EMG training should enable subjects to produce consistent and distinguishable muscle patterns. It is not possible to design a long-term ML algorithm for each subject because it requires a lot of time and resources. Therefore, if users do not perform tests in the laboratory, they may encounter limitations in control flexibility or incorrect movements, which is also considered a common reason for abandoning the use of prostheses (Biddiss and Chau, 2007c; Scheme and Englehart, 2011; Chadwell et al., 2016). If users can understand that their training program may lead to poor actual use, they can immediately adjust the training system to reduce unnecessary frustration and help achieve better electromyographic control. Some studies have established more comprehensive offline training metrics before real-time experiments, including classification measures, variability measures, separability measures, complexity measures, and neighborhood measures (Ortiz-Catalan et al., 2014; Franzke et al., 2021; Nawfel et al., 2021). The classification index is a measure that describes the correctly computed prediction score of the system. The variability metrics is a measure of the reproducibility of EMG patterns between repetitions, which quantifies intra-class characteristics and feedback on the consistency of EMG patterns. The separability metrics is a measure of the reproducibility of EMG patterns between classes, which assesses inter-class characteristics.

**FIGURE 5:**
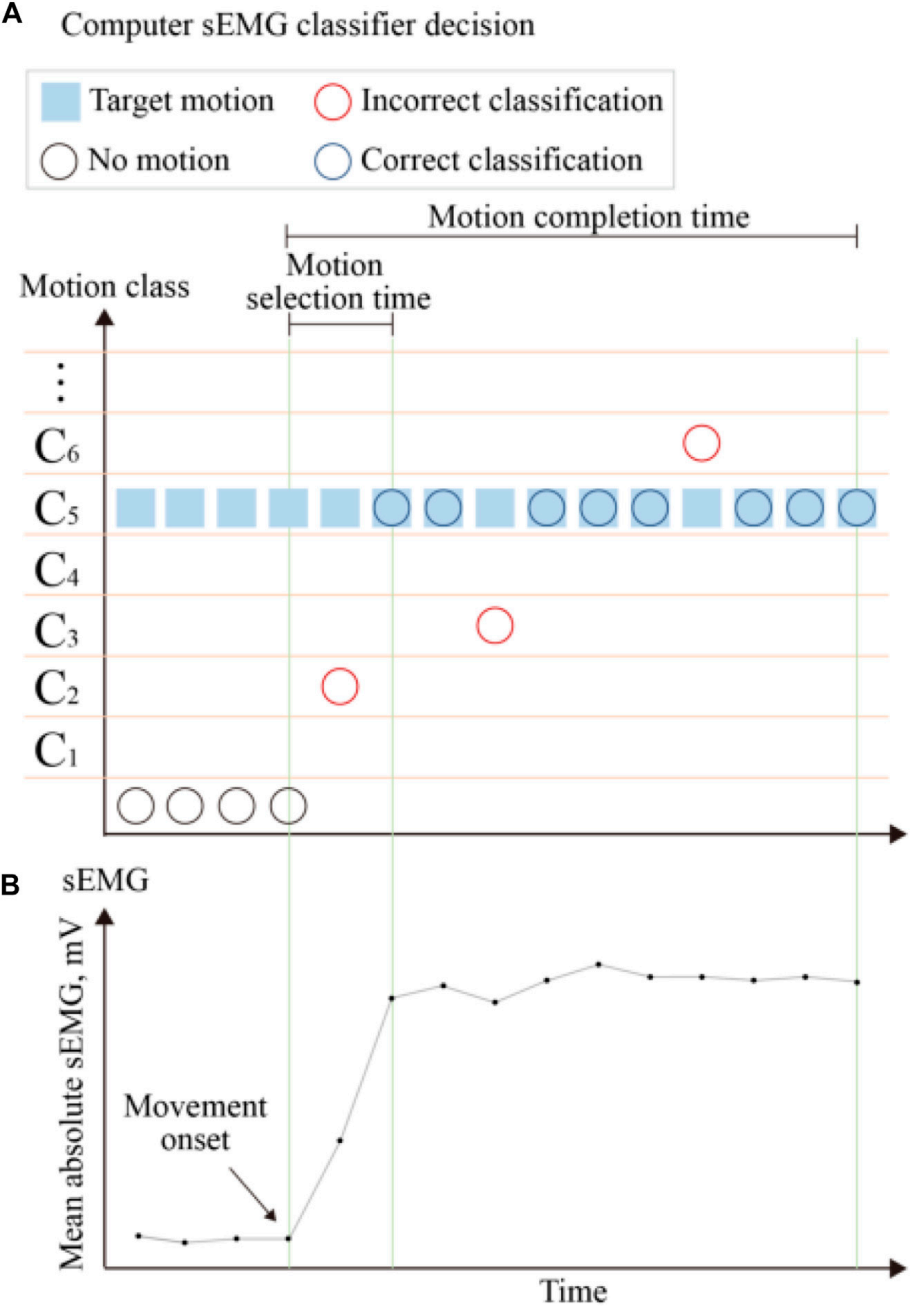
Definition of the real-time performance metrics. The mean absolute values of the sEMG signals used to generate control commands are shown at the **(B)**. The **(A)** present the control decisions generated in response to the sEMG signal classifier and corresponding performance metrics. The blue boxes indicate the target motion. The control decisions are represented by three circles, with black circles indicating decisions with no motion, blue circles indicating decisions with correct classification, and red circles indicating decisions with incorrect classification. The performance metrics shown include motion selection time and motion completion time.

To more comprehensively measure training effect and task difficulty in real-time testing, previous research has proposed many online performance metrics. Most clinical assessments test the ability to perform specific movements using time-related parameters (Joyner et al., 2021), as illustrated in Figure 5. Motion completion time is defined as the time from movement initiation to task completion, which includes the full range of motion of each movement. Motion selection time is the time required to correctly select the target motion, which is used to quantitatively measure the speed at which the motion command is converted into a correct motion prediction. Motion completion rate is the percentage of the total motion attempts that are successfully completed within the time limit. Task attempt is defined as the number of times the subject initiates interaction with the object and moves toward task completion (Bangor et al., 2008). Motion quality is defined as the number of awkward and compensatory movements used by the subject in the process of completing the task.

Based on the fact that users must respond to and correct the system’s misclassifications to successfully complete the task, Fitts’ law, which can be used to demonstrate that any movement task exhibits a trade-off between speed and accuracy, has also been widely used to evaluate online myoelectric control (Fitts, 1954). Fitts’ Law typically uses completion rate, path efficiency, overshoot or throughput, and other parameters to evaluate the online performance of XR systems (Park et al., 2008; Scheme and Englehart, 2013; Gusman et al., 2017; Nawfel et al., 2021). The completion rate was mentioned above. Throughput (TP) is the most important metric in Fitts’ law, which is defined as the transfer of information in the results of repeated tests over different target distances and widths. Path efficiency is defined as the ratio of the shortest path to the actual path to the target. Overshoot is used to count the number of times the target is lost before reaching the stop position during each movement, and to measure the stability of the users’ task performance. The TAC test is very similar to a test based on Fitts’ law, which uses the virtual prostheses on the screen to evaluate the control and positioning ability of the prostheses.

## 4 Existing challenges and future development

The positive results of the XR prosthetic system involve many aspects of the virtual environment. In this section, we will analyze the current status and possible trends of the system in terms of ADL, training modalities, feedback and the relationship between the virtual environment and the physical device to support the implementation of future systems with more effective training capabilities.

### 4.1 ADL

XR prostheses training should focus on two core themes: user engagement and skill transfer from virtual prosthetics to physical prosthetics. A critical element in using XR as a training and rehabilitation tool is the authenticity of the virtual space created. Obviously, XR system can effectively enhance user participation, but for skill transfer, most studies only verify the performance improvement or abstract control in XR system. It seems to be tacitly assumed that the XR system, which uses the same muscle tissue and corresponding EMG signal as the prosthetic task, which can easily be translated into the improvement of prosthetic control. However, previous studies have shown that the emergence of migration phenomenon requires virtual space to be as close as possible to the target of physical prosthetic tasks, that is, more ADL training (Belter et al., 2013; Woodward and Hargrove, 2019).

Virtual space has many potential benefits, including task automation, movement scalability, exercise gamification, environmental security and performance tracking. XR prosthesis system is a powerful tool that can generate or present the properties of all virtual models in interactive tasks, including shape, texture, compliance, and interactive features. Using virtual space to simulate ADL-oriented training is a natural extension of the “real world.” (van Dijk et al., 2016b; van Dijk et al., 2016a). have developed a game to simulate grasping tasks, which augments ADL-relevant information and incorporates the proportional relationship between EMG amplitude and end-effector. This study proved for the first time the transfer effect of using ADL-related information on tasks from XR to myoelectric prostheses, but only when the game was designed to encourage behaviors specific to controlling prostheses. The design of XR prostheses system should pay attention to the balance between game motivation and task functionality (Kristoffersen et al., 2021).

The research on ADL-oriented virtual prosthetic training to improve the daily life performance of amputees has begun to appear. Next, ADL-oriented interventions in virtual space should be close to the real world, including a home-like scenario. Specifically, when designing a virtual system, it is necessary to establish the relationship between ADL tasks and virtual tasks, to specify the information about the relationship between ADL goals and user actions that allows adaptive coordination of these actions, because virtual tasks cannot completely simulate ADL in daily life. It seems that the transfer effect is best evaluated by measuring the performance of ADL tasks, such as the timing of closing or opening the hand, which is also a direction to be improved. The ADL-related training based on actual activities raises an interesting point. In this case, the user would be naturally induced to move and manipulate objects at different heights. This exercise and training performed/exerted by muscles other than the missing ones could form a new physical therapy, which is more conducive to the rehabilitation of users.

### 4.2 Train modalities

The feature of EMG signal are easily affected by external factors, such as muscle fatigue, electrode displacement, limb position change, contraction force change and individual differences. This type of influence cannot be suppressed, and it is also unpredictable. However, it is impractical to account for all the confounding factors in a single training session. Therefore, when myoelectric control is introduced into clinical practice, it is very necessary to have an effective, unified and easy-to-implement training protocol. Putting users’ daily life in the center of research and formulating research objectives, and improving the clinical application performance of the system with clinically relevant results as the goal. The existing research shows that the XR prostheses training system is far behind the new dexterous prosthetic hand and the advanced functional evaluation model. The XR prosthetic training system should be designed by integrating the prosthetic hand control mechanism, such as switch, threshold, proportion, pattern switch and pattern recognition. At the same time, it can integrate multiple functional elements, including training intensity, training times and training level, and even the training level and program required by users (Winslow et al., 2018; Prahm et al., 2019a,8; Prahm et al., 2017c).

Due to technical limitations, XR prostheses system usually only describes the virtual hand on the screen or in a two-dimensional environment, excluding multi-DOF depth of field control and the joint environment with joint drive as the goal, and has no connection with the user’s body. We believe that the future XR prosthetic system should adopt the AR/MR technology combined with IMU, where virtual reconstruction is carried out with the help of IMU tags attached to the user’s body, so the virtual hand or virtual prosthetic will cover the user’s residual limb. The system predicts or tracks the trajectory of the virtual hand through IMU, and controls the virtual hand through the user’s muscle contraction. More realistic virtual hand models, interactive objects and rich scenes would not only provide a unique personalized training interface, but also create a more attractive, more immersive and realistic user experience. Adding game function design, task type, scoring mechanism, type of control scheme used by the program, and feedback can effectively attract users to focus on the results of the game (external focus of attention) rather than on muscle changes (internal focus of attention) during training, which can improve cognitive effort and lead to faster, more accurate, and more effective virtual hand movements.

The game elements are alternated to adapt to the specific needs and development of users at different times or training stages. The virtual system should have a built-in logging capabilities to record the movement status of each component in the virtual space during training and to evaluate the overall rehabilitation performance. The XR prostheses system with rich elements can be used by users to create or select more specific training scenarios. In addition, when combined with accurate rehabilitation methods, it can also provide a higher level of personalized training programs. A better training effect for users would result from a more comprehensive, more clinical and more entertaining virtual prosthesis training.

### 4.3 Feedback

To improve user participation, the existing XR prosthetic system tends to pay more attention to aesthetic design, but ignores functionality. The virtual hand is usually represented by a game element or visualisation. Interacting with objects in the XR environment typically involves attaching them to the hand through programming, rather than controlling the virtual hand using a myoelectric controller that mimics the functionality of the prosthetic hand (Hargrove et al., 2018; Nissler et al., 2019; Phelan et al., 2021) compared the virtual TAC test with a set of outcome measures for physical prostheses, including SHAP, JTHF, BBT, and CRT. Their findings showed a correlation between virtual test measurement and physical performance, but no causal relationship was found. (Boschmann et al., 2021). proposed an AR system that enables users to practice pattern classification control, modulate grasping force with feedback, and adjust wrist rotation via a tilted bar. Through testing, the system can transfer the skills needed to control actual prosthetics. Judging the effectiveness of the training is primarily based on the subjective feedback from the therapist and user, which poses challenges to the objective assessment of the outcomes. The study indicates that the provision of force feedback can enhance the level of realism in the virtual environment and the user’s sensation of embodiment with the virtual hand. Additionally, it can augment the performance of the virtual system and effectively enhance the user’s training outcome (Dosen et al., 2015).

In the straightforward task of grasping, the objective is to lift a cylindrical object with uncertain measurements of diameter, hardness, and friction. The prosthetic hand user must regulate the aperture of the prosthetic hand to correspond with the size of the object, which is essential for skillful utilization of the prosthetic hand. To prevent any breakage of the object, the user must also have the capability to adjust the virtual hand’s force in response to the object’s hardness. Force feedback is essential in virtual environments because users are unable to sense grip force directly. To assist users, a virtual strength can be applied to the object, enabling them to proficiently regulate the force the virtual hand exerts after numerous training sessions. Additionally, friction feedback can be incorporated to simulate objects slipping, providing users with an opportunity to practice all feasible object manipulation strategies.

The development of XR systems would not eradicate work, but rather redistribute and reshape existing activities. Although XR has several advantages over CPT, physiotherapists still play an important role (Almeida and Nunes, 2020). We consider the suggestions of physiotherapists to be a special kind of feedback. The XR prosthetic system and CPT have a mutually advantageous relationship and the program promotes patient engagement while ensuring scientifically-sound training methods. The physical therapist creates a training plan that encompasses a preliminary diagnosis and follow-up assessments. The therapist informs the patient about their condition, adjusts the system, recommends exercises, and assesses the outcomes to attain the anticipated advantages of participation and intervention. Additionally, physical therapists can aid individuals in choosing suitable training methods, difficulty and intensity levels, and tools based on individual traits and interests, creating a personalized training experience. Even when training at home, physical therapists can monitor an individual’s progress through the Internet of Things and take part in their training. Furthermore, it is crucial for physical therapists to be part of the design process of XR systems to ensure their optimization.

### 4.4 Virtual environment and physical devices

The XR prostheses system serves two primary purposes: neuromotor rehabilitation and prosthetic control training. However, the system currently prioritizes neuromotor rehabilitation and virtual prostheses training, disregarding the crucial process of amputees adapting to new prostheses devices (P, 2016). It is necessary to consider these variances when implementing and interpreting results. During clinical practice, XR is mainly used for EMG signal control training to restore muscle function and encourage voluntary muscle contraction. During this time frame, participants practiced grasping objects of different shapes and sizes and performing daily tasks. Subsequently, they received training on how to perform these tasks using a myoelectric prosthetic hand. However, amputees who have achieved voluntary control of EMG signals could potentially face challenges while performing tasks as intended. The impact of prosthetic weight and arm posture on EMG signals, the inconsistencies between XR tasks and actual grasping tasks, and differences in virtual versus real prosthetic hand models contribute to these findings. While some studies suggest that training with virtual prostheses is equivalent to training with physical ones, the extent to which skills acquired in the simulated environment are transferable to the actual task remains unclear.

The virtual prostheses can be programmed and calibrated to replicate the physical prosthetic system, allowing users to practice controlling the system using virtual objects (Lambrecht et al., 2011; Kluger et al., 2019; Elor and Kurniawan, 2020; Chappell et al., 2022) proposed a pre-prosthetic hand training system that integrates virtual reality with a robot arm. This system employs the robot arm to simulate the actions and forces of the virtual arm through precise physical simulation. The study reveals that implementation of robot arms can significantly improve training outcomes. However, a gap between the real and virtual environment increases user frustration. Migrating XR prosthetic systems effectively requires consistency and similarity in their function and training with physical prosthetic systems. While relevant guidelines have been proposed for training, scoring, and clinical interpretation, differences still exist in the selection and completion time of movements, task attempts, task quality, and tolerance required to achieve the target posture.

The use of the XR prostheses system in neuromotor rehabilitation training could lead to more complex and distinguishable EMG patterns compared to movements typically used in activating a prosthetic hand. To optimize training effectiveness, it may be beneficial to limit movements that mimic prosthetic control (Na et al., 2017; Kristoffersen et al., 2020). The virtual reality programming engines, such as Unity and Unreal Engine, have precise physics calculation engines that simulate prosthetic hand movements, mechatronic models, delays, and limitations. This enables the implementation of the same model for virtual space control and physical prosthetics. Thus, virtual prosthetics training can lead to direct transfer for physical prosthetics control. During training, advanced prosthetic technology is utilized to create an intuitive and easily manageable system that combines both virtual and real simulations of prosthetic hands. Furthermore, the XR prostheses system and desktop prosthetic hand or prostheses simulators are combined in order to optimize the training process. The user has the ability to adjust the level of control required for training, thereby facilitating gradual improvement in rehabilitation progress. The system design would provide users with a realistic simulation of a future prostheses, enabling them to perform rehabilitation tasks quickly, similar to their experience with physical prosthetics during the early stages of amputation. Additionally, this feature would empower developers to test and assess the structural design and control performance of the prosthetic hand based on personalized user problems, thereby improving the hands’ adaptability.

## 5 Conclusion

This review presents recent advances in XR systems applied to myoelectric prostheses, including existing XR prosthetic systems, virtual control methods, performance evaluation methods, and performance metrics. Our analysis of XR prosthetic systems indicates that serious games can increase user engagement, while simulated tasks improve training outcomes. Existing systems have achieved satisfactory training outcomes, while performance evaluation methods and metrics are continually undergoing refinement. In addition to enhancing user engagement, the XR prosthetic system can serve as a pre-training tool during the wait for a new prosthesis. There are limited direct strategies for transferring performance from virtual environments to physical devices in current systems. However, the emergence of AR/MR technology seems to address this issue. To achieve this objective, this paper compares prosthetic applications, identifies gaps in virtual control methods, performance assessment methods, and physical prosthetic systems, and analyzes the limitations of existing systems while examining proposed development prospects in four areas: ADL, training modalities, feedback, and the relationship between the virtual environment and the physical device. The application of XR technology for myoelectric prosthetic hand training and rehabilitation undoubtedly holds great promise. Establishing a patient-centered XR prosthetic system that is aimed at and inspired by real-world use cases is essential for surmounting hurdles to adoption.
